# Supplementary material for: Medical face masks offer self-protection against aerosols: An evaluation using a practical in vitro approach on a dummy head
Source: PLoS One. 2021 Mar 3;16(3):e0248099. doi: 10.1371/journal.pone.0248099 (PMC7928437; doi:10.1371/journal.pone.0248099)
Supplement: S1 File — (PDF) [file pone.0248099.s001.pdf]

Supplement 1

Tested Masks and Respirators

mask	manufacturer	product	LOT	certification
# 25 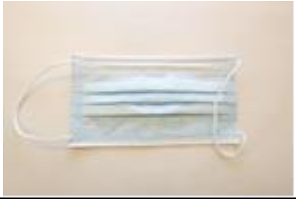	MEDLINE	FACE MASK – WITH EARLOOPS	LOT: 20CASP096 REF NON27373	non-certified
# 26 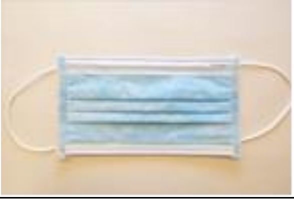	Medi-Inn +	Mundschutz - 3-lagig-blau	LOT: E2011891	EN14683 Typ II, CE-Zertifizierung
# 32 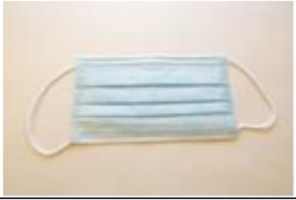	Yeso-med®	Face Mask For Single Use	LOT: 2003042	non-certified
# 1037 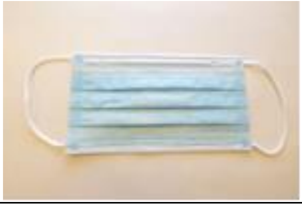	IMD®-Innovation For You	Disposable Medical Face Mask (Non-Sterile)	LOT: 603424	non-certified
# 1042 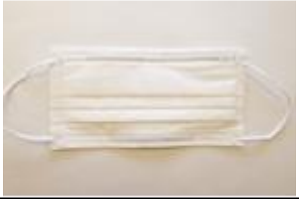	CROSSTEX®-A CANTEL MEDICAL COMPANY	Ultra® Sensitive FOGFREE™	REF GCFCXSFSF	- EN14683 Type IIR Standard<br>- ASTM F2100-11 Level 3<br>- CE
# braun 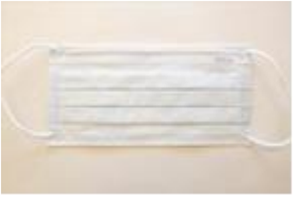	BRAUN	Folitex® Earloop	REF 6073311 LOT0801CLP20	EN14683 Type II CE
# kohle 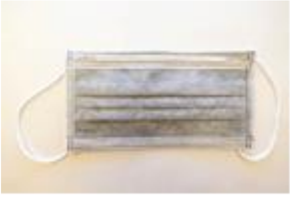	HYOGOSTAR	PP-Mundschutz mit Aktivkohle - 3-lagig	LOT: 972267190021024	non-certified

# Zum Binden<br>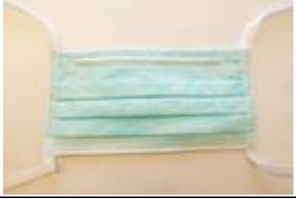	BARRIER	Surgical<br>Mask	REF 42290<br>LOT 20390000	EN:14683 Typ II<br>CE
# Zum Binden gestreif<br>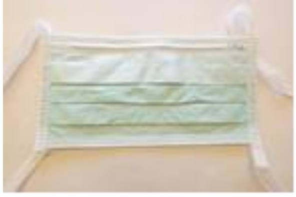<br>t	Mölnlycke	Barrier®<br>Medical<br>Face Mask<br>Special	REF 4238<br>LOT 20140000	EN:14683:2019<br>+AC:2019 Type II<br>CE
# 6<br>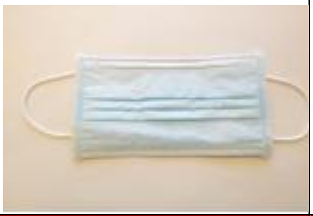	JOINTOWN	FACE MASK<br>with Ear<br>Loops	LOT: 2020.03	non-certified
FFP 2				
# 3M<br>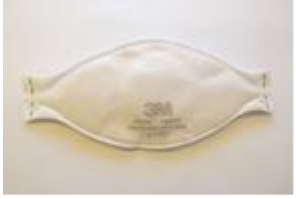	3 M™	AURA ™<br>1862+	C 200951<br>(ESA+ESB+MTA<br>)<br>C202721<br>(ESC+MTB+MT<br>C)<br>C202821<br>(MTD)	EN14683:2005 IIR<br>EN149:2001 FFP2 NR<br>D<br>CE2797
# Moldex<br>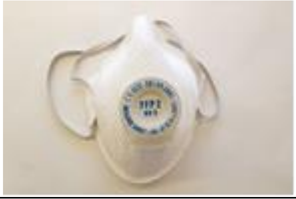	MOLDEX	MOLDEX<br>2405+		EN149:2001<br>CE0121<br>TPTC 019/2011<br>EAC
# CROSSTEX<br>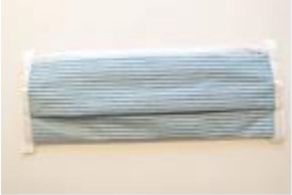	CROSSTEX®-A<br>CANTEL<br>MEDICAL<br>COMPANY	Isolator®<br>Plus	LOT: 1915681	In Reference to<br>EN149: 2001 FFP2 NR<br>CE 0121<br>CDC guidelines for TB<br>exposure control<br>NIOSH approved as<br>an N95 respirator
#3 M Ventil<br>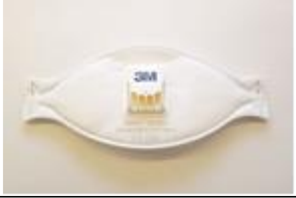	3 M™	AURA ™<br>9322 +		CE 0086 EN 149.2001<br>+A1:2009 FFP2 NRD

# Dräger 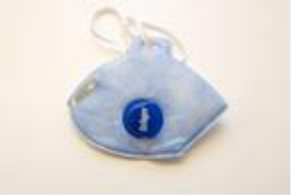	Dräger	X-plore 1720 V FFP2 NRD	VRNF-F024	CE0158 EN 149:2001
Kn95				
#204 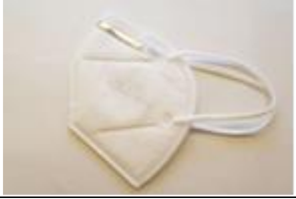	STARBUSS	3D NANO FACIAL PROTECTIVE MASK	Production Batch: 05022020 Date of Manufacture: 05/02/2020	GB 2626-2006
#1013 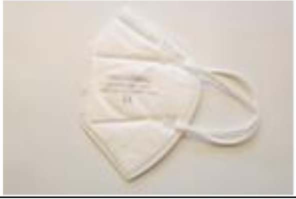	TAIDAKANG™	KN95 Protective Mask (Non Medical)	LOT:R20200423	GB 2626-2006
# Test 5 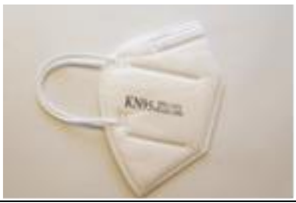	AILEBAO	KN95 PROTECTIVE MASK		GB 2626-2006
#MASK 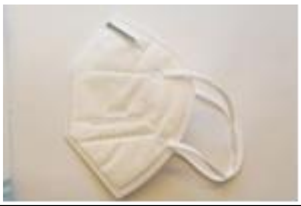	MASK – DISPOSABLE PROTECTIVE	PROTECTIVE MASK		GB 2626-2006
#1016 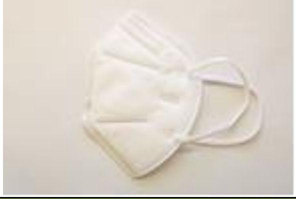	DADDY'S CHOICE®	Purism KN95 Protective Face Mask	2020/05/01	GB 2626-2006
Cloth masks				
#Tr 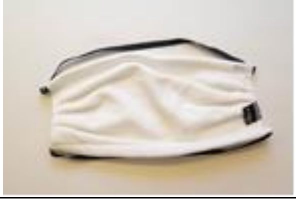	Trigema	N/A	N/A	non-certified

# Filterspezial<br>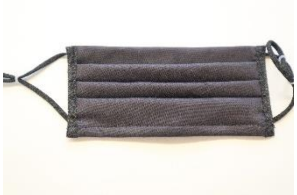	N/A	N/A	N/A	non-certified
#Nano Silver FFP2<br>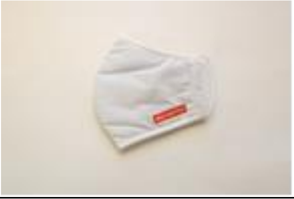	N/A	N/A	N/A	non-certified
#Maske Flieder<br>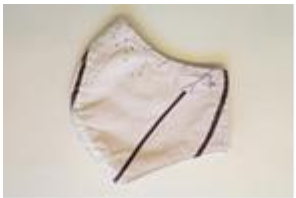	N/A	N/A	N/A	non-certified
#Polycotton<br>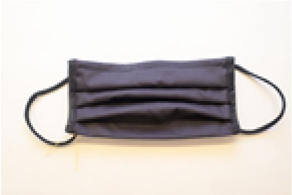	N/A	N/A	N/A	non-certified
#weiß<br>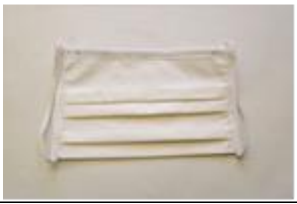	N/A	N/A	N/A	non-certified
#Rose<br>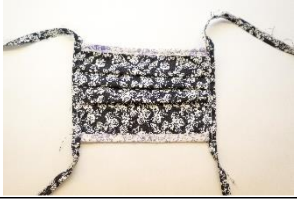	N/A	N/A	N/A	non-certified
#Bunt gefleckt<br>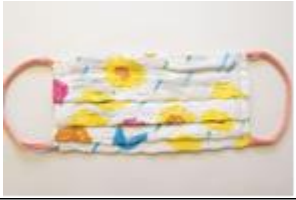	N/A	N/A	N/A	non-certified
#VL<br>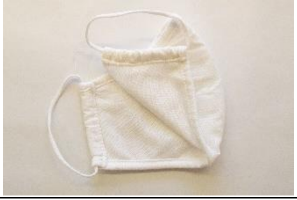	Van Laack	N/A	N/A	non-certified
