# Supplementary material for: Medical face masks offer self-protection against aerosols: An evaluation using a practical in vitro approach on a dummy head
Source: PLoS One. 2021 Mar 3;16(3):e0248099. doi: 10.1371/journal.pone.0248099 (PMC7928437; doi:10.1371/journal.pone.0248099)
Supplement: Attachment — Submitted filename: Review PONE-D-21-01928.docx [file pone.0248099.s002.docx]

This manuscript studied if it is advised to use face masks to protect the human wearer by comparing respirators, medical face masks, and cloth masks. The filtration efficacy and breathing resistance of the mask material were determined. The overall filtration efficiency of simulating real live conditions on a dummy head was also compared. The topic is interesting and urgent under the COVID-19 pandemic. However, a few questions should be addressed and the reviewer would appreciate further discussion in the manuscript. The reviewer recommends a major revision of this manuscript before its publication.

General comments:

1.Lack of information
1) The authors stated that 32 different face coverings were tested. Please list details of the materials information in the Table (e.g., name, brand, materials, etc.), as the results are likely to vary widely among the various brands or models.
2) Page 11, Methods section, Line 11, the fit factor was mentioned in this sentence but did not introduce throughout the paper.

2. Reference

1) Page 11, Methods section, Line 15, some references are necessary to sustain the skin-like surface and the techniques of coated with liquid gum.
2) Page 16, Discussion, Line 16-18, “The chosen airflow rate of 28.3 L/min in our experiments provides a realistic test condition considering a minute volume of 18 L/min and an inspiration/expiration ratio of 1:2”, some related references should be taken into account to support the selected data.

3. Figures

1) Please improve the quality of the figures.
2) Page 14, Overall filtration efficiency section, Line 22-25, as mentioned “figure 3A and figure 3B” here, but the following figure 3 does not contain “A” and “B”.
3) Page 15, Overall filtration efficiency section, Line 4-8, figure 4 is good and essential to indicate the tested results to be seen, but only a few explanations are given in figure 4. The authors also need to illustrate the meaning of lines in this figure.

Detailed comments:

1.Page 13, Material properties section, Line 19, the authors showed the efficiency of cloth masks and non-EN-certified face masks. Please clarify what these efficiencies are: average efficiency or specific efficiency among the tested materials?
2.Page 14, Overall filtration efficiency section, Line 6-10, why are the results of the material test and dummy head testing not matched? Any potential explanations should be suggested by the authors.
3.Page 16, Discussion, Line 14, “With this stable aerosol, it is possible to challenge masks with small particles sized ≤0.5 µm”, why the particles sizes below 0.5 µm are not taken into consideration in the paper since the aerosols can be generated? In addition, SARA-CoV-2 particles are only 60-140 nm in diameter.
